# Melatonin suppresses cisplatin-induced nephrotoxicity via activation of Nrf-2/HO-1 pathway

**DOI:** 10.1186/1743-7075-10-7

**Published:** 2013-01-12

**Authors:** Ulkan Kilic, Ertugrul Kilic, Zeynep Tuzcu, Mehmet Tuzcu, Ibrahim H Ozercan, Okkes Yilmaz, Fikrettin Sahin, Kazim Sahin

**Affiliations:** 1 Department of Medical Biology, Faculty of Medicine, Bezmialem Vakif University, Adnan Menderes Bulvarı Vatan Caddesi, Fatih, TR-34093, Istanbul, Turkey; 2 Department of Physiology, Faculty of Medicine, Medipol University, Istanbul, Turkey; 3 Department of Biology, Faculty of Science, Firat University, Elazig, Turkey; 4 Department of Pathology, Faculty of Medicine, Firat University, Elazig, Turkey; 5 Department of Genetics and Bioengineering, Yeditepe University, Istanbul, Turkey; 6 Department of Animal Nutrition, Faculty of Veterinary Science, Firat University, Elazig, Turkey

**Keywords:** Nephrotoxicity, Nrf2/HO-1 signaling, Melatonin, Oxidative stress

## Abstract

**Background:**

Cisplatin, one of the most effective and potent anticancer drugs, is used in the treatment of a wide variety of both pediatric and adult malignancies. However, the chemotherapeutic use of cisplatin is limited by its serious side-effects such as nephrotoxicity and ototoxicity. Cisplatin chemotherapy induces a reduction in the antioxidant status, leading to a failure of the antioxidant defense against free-radical damage generated by antitumor drugs. Cisplatin-induced oxidative stress in the kidney was partially prevented by antioxidant treatments using superoxide dismutase, glutathione, selenium and flavonoids. Melatonin and its metabolites possess free-radical scavenging activity and it has been shown that they protect against cisplatin toxicity. However, the mechanism of the protective effects of melatonin against cisplatin-induced nephrotoxicity is still essentially unknown. We therefore designed this study to investigate the underlying mechanism of the protective effect of melatonin against cisplatin-induced renal damage in a rat nephrotoxicity model in vivo.

**Methods:**

Twenty eight 8-week-old male Wistar rats were divided into four groups of control, melatonin treatment (4 mg/kg b.w i.p. for 10 days), cisplatin treatment (7 mg/kg b.w., i.p.) and melatonin and cisplatin combination treatment. Serum urea nitrogen (urea-N) and creatinine levels were measured. Histopathological changes were evaluated. In addition, we analyzed the expression levels of HO-1, Nrf2, NF-κB and AP-1 in Western blot analysis.

**Results:**

Both serum creatinine and urea nitrogen increased significantly following cisplatin administration alone; these values decreased significantly with melatonin co-treatment of cisplatin-treated rats. Histological analysis showed that cisplatin caused damage in the proximal tubular cells in the kidneys of cisplatin-treated rats; these changes were reversed by melatonin co-treatment. Upon Western blot analysis, melatonin treatment increased Nrf2 accumulation in the nuclear fraction, and increased the expression of HO-1 in the cytosolic fraction as compared to the cisplatin-treated rats. Expressions of NF-κB p65 and AP-1 were increased significantly in the kidneys of rats treated with cisplatin compared with the expression in the kidneys from the control, melatonin-only-treated and melatonin co-treated rats.

**Conclusion:**

Our present data suggest that melatonin attenuates cisplatin-induced nephrotoxicity possibly by modulating Nrf2/HO-1 signaling.

## Background

Cisplatin (cis-diamminedichloroplatinum) is a widely used antineoplastic drug for the treatment of various cancer types [1,2]. However, its use is limited by its nephrotoxicity with about 25–35% of patients experiencing a significant decline in renal function after a single dose of cisplatin treatment [3]. Recent studies showed that cisplatin induce DNA adduct formation, leading to aberrant genetic transcription and DNA duplication, cell cycle arrest, and induction of apoptosis [4]. Further, increased generation of reactive oxygen species (ROS), which are often involved in renal dysfunction, has been reported in cisplatin-induced nephrotoxicity [5-8]. The mechanism of cisplatin-induced nephrotoxicity is not completely understood; however, several mechanisms, including hypoxia, free radicals, inflammation, and apoptosis are thought to be involved. Excessive production of free radicals, such as superoxide anion, hydrogen peroxide, and hydroxyl radicals, and the occurrence of lipid peroxidation due to oxidative stress are associated with cisplatin-induced renal dysfunction [9,10].

In many cell types, numerous cellular responses to oxidative stress have been found to be involved in signaling proteins that act through the antioxidant response element (ARE) and the transcription factor, the nuclear factor erythroid 2-related factor 2 (Nrf2) [11]. Nrf2, is a redox-sensitive transcription factor, which mainly regulates transcriptional activation through the ARE [12,13]. Under physiological conditions, cytosolic Nrf2 is inactive by its negative regulator Kelch-like ECH-associating protein 1 (Keap1). When cells are exposed to redox modulators, Nrf2 is released from Keap1, translocates and accumulates in the nucleus. Nrf2 forms a heterodimer with a small Maf protein and c-jun [14]. It has been recently documented that as Nrf2 activators various compounds such as polyphenols (curcumin and resveratrol), sulfur-containing compounds (isothiocyanate sulforaphane, phenethyl isothiocyanate), terpenoids (cafestol and kahweol), carotenoids (β-carotene), and selenium induce the expression of cytoprotective protein in an ARE-dependent manner [15].

Redox sensitive molecular targets including transcription factors nuclear factor-κB (NF-κB) and activator protein 1 (AP-1) contain highly conserved cysteine residues and their oxidation, and nitration are essential in the oxidant/redox signaling. Both NF-κB and AP-1 are activated by various physiological and pathological stimuli including ROS directly or their generation through mitochondria [16] and orchestrate expression of many genes playing roles in inflammation, embryonic development, lymphoid differentiation, oncogenesis, and apoptosis [17].

Melatonin (N-acetyl-5-methoxytriptamine) is synthesized and released into the circulation and especially into cerebrospinal fluid by the pineal gland in a circadian rhythm [18] and is also produced by immune system cells, brain, airway epithelium, bone marrow, gut, ovary, testes, skin and likely other tissues [19]. Melatonin and its metabolites possess free-radical scavenging activity [20,21]. Melatonin has both receptor-mediated and receptor independent actions and is believed to affect all cells [22,23]. Melatonin increases mRNA and protein levels of antioxidant enzymes through Nrf2 activation [24,25]. Negi et al. (2011) reported that melatonin ameliorates neuroinflammation and oxidative stress through Nrf2 and NF-κB in experimental diabetic neuropathy. Upregulation of Nrf2 by melatonin resulted in an increased expression of antioxidant enzyme heme oxygenase-1 (HO-1) [26].

Cisplatin-induced oxidative stress in kidneys was partially prevented by antioxidant treatments using superoxide dismutase, glutathione, selenium and flavonoids [27]. Melatonin has been shown to protect against cisplatin toxicity [28,29]. However, the mechanism of the protective effects of melatonin against cisplatin-induced nephrotoxicity is still essentially unknown. We therefore designed this study to investigate the mechanism of the protective effect of melatonin against in vivo cisplatin-induced renal damage in a rat nephrotoxicity model.

For this purpose, serum urea nitrogen (urea-N) and creatinine levels were measured. Histological changes were evaluated and the expression levels of HO-1, Nrf2, NF-κB and AP-1 were analyzed in Western blot analysis.

## Experimental methods

### Animals

Male Wistar rats (n = 28, 8 wk-old), weighing 200–215 g, were obtained from Firat University Research Center (Elazig, Turkey). The rats were kept in an environmentally controlled room at constant temperature (21 ± 1°C) and humidity (75 ± 5%) under a 12 h light/dark cycle. The animals were acclimatized for 1 week before the study and had free access to standard laboratory feed and water ad libitum. The study has the permission of Ethics Review Committee for Ethics in Animal Experiments of the Firat University and guidelines for the Care and Use of Laboratory animals were strictly followed.

### Experimental protocol

Kidney injury was induced by a single intraperitoneal (i.p.) injection of cisplatin (Sigma Chemical Co, USA) (7 mg/kg b.w.) [30]. Twenty-eight 8-week-old male Wistar rats were divided into four groups of control treated with vehicle, melatonin-treated (4 mg/kg b.w, i.p. at 17:00 hr. for 10 days) [31], (Sigma-Aldrich, St Louis, MO, USA), cisplatin treated (7 mg/kg b.w., i.p.), and melatonin (4 mg/kg b.w., i.p. at 17:00 hr. for 10 days) and cisplatin (7 mg/kg b.w., i.p.) co-treated.

Melatonin administration was started two days before the single i.p. injection of cisplatin. Melatonin was dissolved in ethanol and diluted in saline. Final ethanol concentration was 1%. On day 12 (10 days after the cisplatin treatment), all rats were sacrificed by cervical dislocation under anesthesia (1% Halothane). Blood samples were taken for serum analyses and the kidneys were removed for histological studies and Western blot analysis.

### Biochemical measurement

Blood samples were centrifuged at 3.000 *g* for 10 min, and sera were collected. Serum urea nitrogen (urea-N) and creatinine were measured using biochemical analyzer (Olympus AU-660, Osaka, Japan).

### Western blot analysis

Protein extraction was performed by homogenizing the rat kidneys in 1 ml ice-cold hypotonic buffer A, containing 10 mM HEPES (pH 7.8), 10 mM KCl, 2 mM MgCl_2_, 1 mM DTT, 0.1 mM EDTA, and 0.1 mM phenylmethylsulfonyl-fluoride (PMSF). To the homogenates 80 μl of 10% Nonidet P-40 (NP-40) solution was added, and the mixture was centrifuged for 2 min at 14,000 g. Supernatant containing the cytosolic fraction was collected for HO-1. The precipitate containing the nuclear fraction was separated for Nrf2, NF-ĸB-65 and AP-1, washed with 500 μl of buffer A plus 40 μl of 10% NP-40, centrifuged, resuspended in 200 μl of buffer C [50 mM HEPES (pH 7.8), 50 mM KCl, 300 mM NaCl, 0.1 mM EDTA, 1 mM DTT, 0.1 mM PMSF, 20% glycerol], and centrifuged for 5 min at 14,800 *g*. The supernatant from the abovementioned precipitate was collected for Nrf2, NF-ĸB p65 and AP-1. Protein concentrations were determined according to the procedure described by Lowry using a protein assay kit supplied by Sigma (St. Louis, MO, USA). Sodium dodecyl sulfate–polyacrylamide gel electrophoresis sample buffer containing 2% b-mercaptoethanol was added to the supernatant. Equal amounts of protein (50 μg) were electrophoresed and subsequently transferred to a nitrocellulose membrane (Schleicher and Schuell Inc., Keene, NH, USA). Blots on the nitrocellulose membrane were washed twice for 5 min each in PBS and blocked with 1% bovine serum albumin in PBS for 1 h prior to the application of the primary antibody. Antibodies against Nrf2, HO-1, NF-κB p65 and AP-1 were purchased from Abcam (Cambridge, UK). Primary antibody was diluted (1:1000) in the same buffer containing 0.05% Tween-20. The nitrocellulose membrane was incubated overnight at 4°C with primary antibody. The blots were washed and incubated with horseradish peroxidase-conjugated goat anti-mouse IgG (Abcam, Cambridge, UK). Specific binding was detected using diaminobenzidine and H_2_O_2_ as substrates. Protein loading was controlled using a monoclonal mouse antibody against ß-actin (A5316; Sigma). Bands were analyzed densitometrically using an image analysis system (Image J; National Institute of Health, Bethesda, USA).

### Histological analysis

The left kidney from each animal was immediately fixed in 20% neutral buffered formalin solution for histopathology. Kidneys were gradually dehydrated, embedded in paraffin, cut into 5-*μ*m sections, and stained with hematoxylin and eosin for histological examination according to standard procedure [32]. Histological changes were evaluated semiquantitatively by a pathologist unaware of the type of treatment. A minimum of 10 fields for each kidney slide was examined and assigned for severity of changes using the following scale: −, none; +, mild damage; ++, moderate damage; and +++, severe damage.

### Statistical analysis

Sample size was calculated based on a power of 85% and a p-value of 0.05. Given that assumption, a sample size of 7 per treatment was calculated. The data were analyzed using the GLM procedure of SAS (2002). The treatments were compared using ANOVA and p < 0.05 was considered statistically significant. Inter-group differences in latencies were determined by the analysis of variance for repeated measurements (ANOVAR) followed by Fisher’s post hoc test for all groups.

## Results

### Biochemical measurement

Both serum creatinine and urea nitrogen increased significantly following cisplatin administration alone; these values decreased significantly with melatonin co-treatment of cisplatin-treated rats (Table 1).

**Table 1:** The effect of melatonin administration on urea-N and creatinine levels in kidney of experimental rats (n=10)

**Item**	**Groups**			
	**Control**	**Melatonin**	**Cisplatine**	**Melatonin + Cisplatine**
Urea-N (mg/dl)	31.5 ± 3.4^c^	30.9 ± 2.0^c^	438.9 ± 63.1^a^	209.0 ± 42.5^b^
Creatinine (mg/dl)	0.68 ± 0.19^c^	0.67 ± 0.14^c^	3.61 ± 0.43^a^	1.91 ± 0.57^b^

Values are mean ± SE of 10 rats from each group. a, b, c: means in the same row not sharing a common superscript are significantly different (*P*< 0.05) between groups.

### Western blot analysis

**Figure 1:**
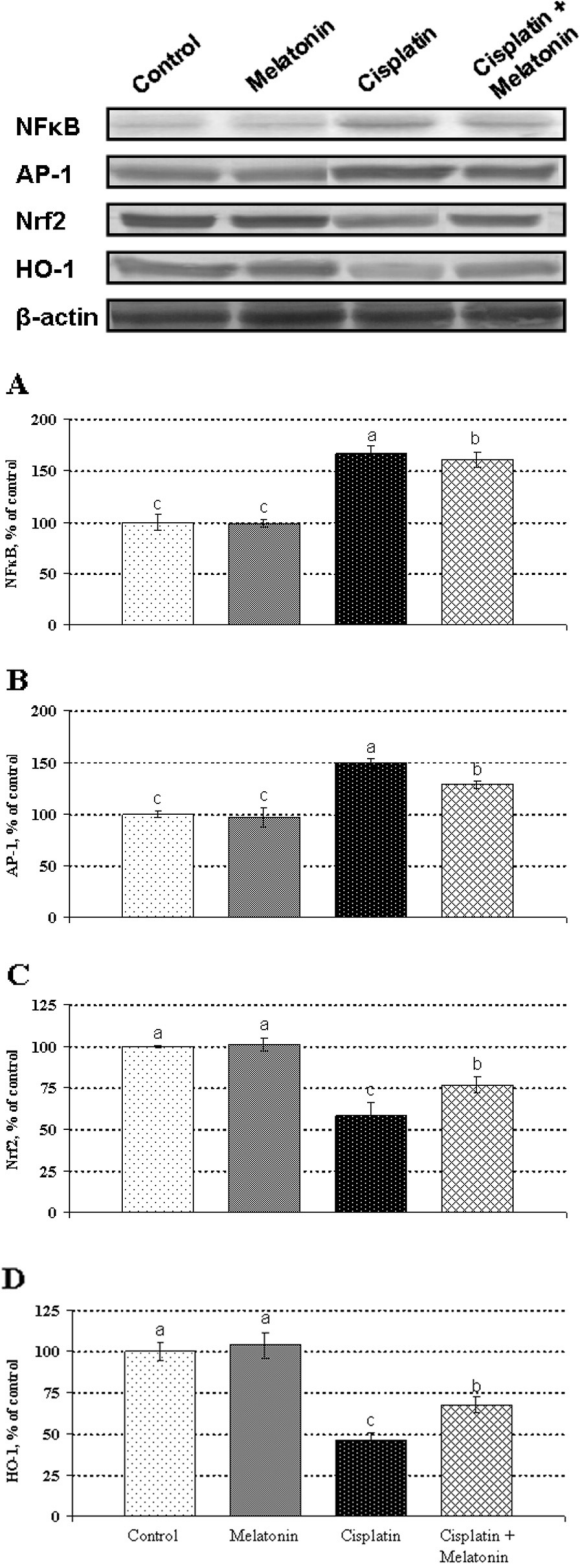
**Western blot analysis of NF-κB p65, AP-1, Nrf2 (nuclear fraction) and HO-1 (cytosolic fraction) in kidney cells in rats: Western blot using the anti- NF-κB (Panel A), AP-1 (Panel B), Nrf2 (Panel C) and hemeoxygenese-1 (HO-1; Panel D) revealed specific bands.** Blots were repeated at least 3 times. β-actin levels were monitored to ensure equal protein loading (bottom panel). The intensity of the bands was quantified by the densitometric analysis. Data are percent of the control. a-c: Means in the same line without a common superscript differ significantly (P < 0.05).

Expressions of NF-κB p65 and AP-1 were increased significantly in the kidneys of rats treated with cisplatin compared with the expression in the kidneys from the control, melatonin-only-treated and melatonin co-treated rats (*P* < 0.05) (Figure 1). In Figure 1 it was shown that melatonin treatment increased Nrf2 accumulation in the nuclear fraction (*P* < 0.05), and increased the expression of HO-1 in the cytosolic fraction as compared to the cisplatin-treated rats (*P* < 0.05).

### Histological analysis

**Figure 2:**
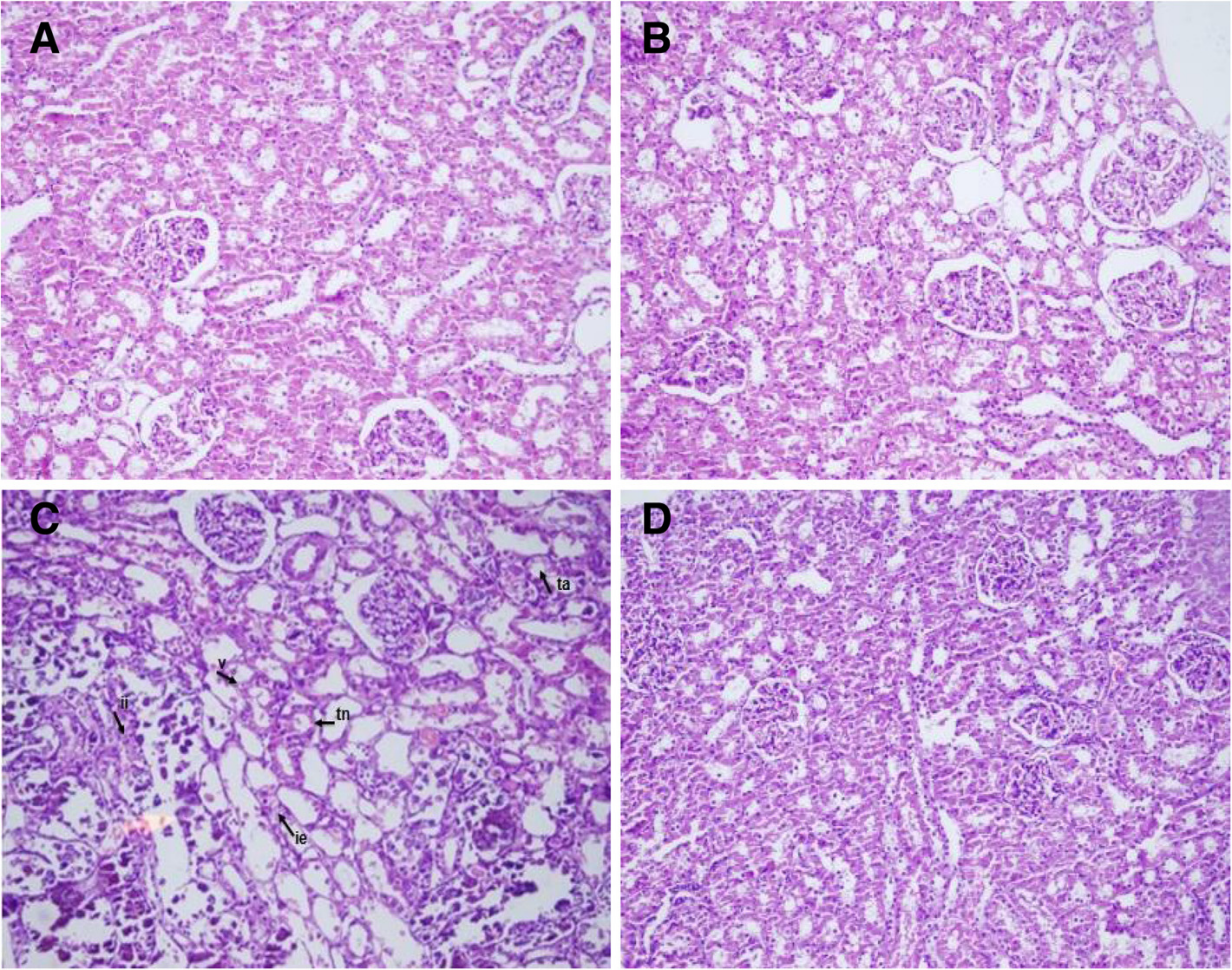
**Histological changes in renal tissues in response to cisplatin and cisplatin+melatonin: The day when animals injected cisplatin is Day 0 and the histological changes in the renal tissues on day 10 are indicated. A**, Control; **B**, melatonin treatment alone; **C**, cisplatin treatment alone [left to right (ii) interstitial inflammation, (v) vaculation, (ie) interstitial edema, (tn) tubular necrosis, (ta) tubular atrophy]; **D**, cisplatin+melatonin. Magnification: x 200.

The kidneys from the control rats and the rats treated with melatonin only showed no abnormality, whereas the kidneys from the cisplatin-treated rats showed marked histological changes in the cortex and outer medulla, such as vacuolation (v), interstitial edema (ie), tubular atrophy (ta), severe tubular necrosis (tn), and interstitial inflammation (ii). Melatonin treatment decreased the cisplatin-induced tubular necrosis and most of the changes were caused by cisplatin treatment (Figure 2, Table 2).

**Table 2:** **The effect of melatonin administration on morphological changes as assessed by histological analysis of kidneys in rats (*n *= 10)**

	**Groups**			
**Changes**	**Control**	**Melatonin**	**Cisplatine**	**Melatonin + Cisplatine**
Vaculation	-	-	++	+
Interstitial edema	-	-	+	-
Tubular brush border loss	-	-	+	+/−
Tubular necrosis	-	-	++	+
Tubular atrophy	-	-	+	+/−
Interstitial inflammation	-	-	+++	+/−

−, none; +, mild damage; ++, moderate damage; and +++, severe damage are semiquantitative scores given by a pathologist unaware of the type of treatment according to method of Ross et al. (1989).

## Discussion

The present study demonstrates that the administration of melatonin exerts a renal protective effect in a rat model of nephrotoxicity provoked by a single injection of cisplatin. We analyzed expressions of HO-1, Nrf2, NF-κB and AP-1 in Western blot analysis. The expressions of Nrf2 and HO-1 were increased significantly. Expressions of NF-κB p65 and AP-1 were increased significantly in the kidneys of rats treated with cisplatin compared with the expression in the kidneys from the control, melatonin-only-treated and melatonin co-treated rats.

Both serum creatinine and urea nitrogen increased significantly in cisplatin treated animals; however, these effects of cisplatin reversed by melatonin treatment. Histological analysis showed that cisplatin damaged the proximal tubular cells; these changes were prevented by melatonin co-treatment. Melatonin alone did not show any significant effect on NF-κB, AP-1, Nrf2 and HO-1 in the kidneys of animals without cisplatin treatment. In previous studies, it has been shown that cisplatin enhances the production of ROS, decreases the antioxidant enzyme levels, enhances the level of TNF-α [33], and induces apoptosis [34] while triggering its toxicity. ROS generated by cisplatin are crucial for Nrf2-driven transcriptional activation of ARE. This led us to expect that cisplatin might induce nuclear translocation of Nrf2, and activate NF-κB; NF-κB activation by ROS has been reported in a previous study [35]. Nrf2 is a basic leucine zipper transcription factor, which transcriptionally regulates many genes including HO-1, NAD(P) H:quinine oxidoreductase-1, c-glutamylcysteine synthase, and glutathione S-transferase [36]. The inactive form of Nrf2 is localized in the cytoplasm bound to a cytoskeleton-associated protein, Keap1. Its activation is considered to be an important molecular target of many chemopreventive and cytoprotective agents [37-39]. Nrf2 protects the cell against oxidative stress through ARE-mediated induction of several phase 2 detoxifying and antioxidant enzymes, particularly the HO-1 [38-40]. HO-1 is a stress-responsive enzyme, responsible for the breakdown of heme to biliverdin, free iron and carbon monoxide [36,41]. It is induced by a variety of cellular stresses, including heme, hyperoxia, hypoxia, and electrophiles [36,39]. Beni et al., (2004) reported that the activation of transcription factor Nrf2 influenced by the cell redox, which acts as a sensor of electrophiles and prooxidant stressors [42]. Oxidative stress and inflammation are two of the most critical factors implicated in cisplatin-induced nephrotoxicity. While Nrf2 upregulates the expression of a number of antioxidant proteins, role of ROS in inflammation via the activation of NF-kB has been investigated in cisplatin-induced renal injury [43]. Recent studies have shown the interaction between nuclear Nrf2 and NF-κB signaling [24,36]. The studies also report that Nrf2-deficient mice display increased NF-κB activation in response to lipopolysaccharides [44]. Furthermore, disruption of Nrf2 enhances the upregulation of NF-κB and proinflammatory cytokines in the brain after traumatic brain injury [36,45]. Cisplatin treatment showed an inverse correlation between the two transcriptions factors, which is in agreement with previous studies.

Melatonin (N-acetyl 5-methoxytryptamine) and its metabolites have been shown to enforce the antioxidant system by scavenging free radicals [21,46]. Melatonin stimulates synthesis of antioxidant enzymes [47] and increases the activities of other antioxidants [48,49]. Furthermore, melatonin protects antioxidative enzymes from oxidative damage [50]. Wang et al., (2009) previously showed that melatonin was effective in preventing cardiopulmonary by-pass-induced renal damage probably through its antioxidant function and upregulation of HO-1 [51]. The indole, melatonin, is well tolerated, has a low interaction potential with other medications and in some cases may even reduce the side effects of synthetic drugs because of its free-radical scavenging properties [52]. We and others have previously shown that melatonin, which based on its small molecular size and high lipophilicity, possesses excellent biological membrane permeability and minimal side effects in humans [18,23,53], reduces brain injury in mouse [23,54-56] and rat [57,58] models of ischemic stroke.

Melatonin as an antioxidant has been investigated in various animal models such as age related neurodegeneration [59], in traumatic brain injury [44], left ventricular hypertrophy [60] and antibiotic-induced nephrotoxicity such as anthracyclin antibiotics, and gentamicin [61,62] and in different nephrotoxic models [63,64]. In the last decade, in various models of acute and chronic tissue injury and oxidative stress, it has been shown that the main mechanism for melatonin’s protective effect is its action through indirect (transcriptional) effects. It has been recently shown that the melatonin-derived protection of heart damage caused by acute exercise in rats is associated with the NF-κB dependent control of inflammatory and pro-oxidant pathways [65,66]. In a model of acute renal damage in rats, melatonin was found to improve markers of oxidative stress by increased expression of the antioxidant and detoxification enzyme HO-1 [50] or by inhibition of the inducible form of NOS and also of p38 MAPK and NF-κB activation [67].

Beni et al. (2004) reported that AP-1 transcription factor inhibition by melatonin played an important role in the late protection response to traumatic brain injury [42]. Hepatoprotective effects of melatonin were demonstrated in rats after acute intoxication with dimethylnitrosamine, which provides further support to a role for melatonin as a secondary antioxidant and detoxification agent [66]. The mechanism seems to be related to negative modulation of NF-κB -dependent genes activated in response to stress.

## Conclusion

In conclusion, the present study was carried out to investigate the expressions of Nrf2, HO-1 NF*κ*B and AP1 after a single cisplatin injection in rats. We evaluated the possible prevention of cisplatin-induced oxidative stress in the kidney with melatonin administration. Here, we report that melatonin attenuates cisplatin-induced nephrotoxicity in rats by modulating Nrf2/HO-1 signaling. Expressions of NF-κB p65 and AP-1 were increased significantly in the kidneys of rats treated with cisplatin compared with the expression in the kidneys from the control, melatonin-only-treated and melatonin co-treated rats. Nrf2/HO-1 signaling pathway upregulates the expression of a number of antioxidant genes in response to a wide array of stimuli, and protects the cell against oxidative stress and inflammation [39]. The results of this study indicate a possible association between Nrf2/HO-1 antioxidative stress signaling and melatonin’s nephroprotective effect.

These data may have research hints at therapeutic uses for melatonin. Melatonin may be beneficial in the prevention of cisplatin-induced nephrotoxicity. However, additional clinical studies are needed to evaluate the role of preventive melatonin treatment in humans. The mechanisms of melatonin’s effect in terms of Nrf2/HO-1 regulation including some other transcription factors could be further investigated.

## Competing interests

The authors declare that there are no competing interests.

## Authors’ contribution

The project was designed and implemented by UK and KS. Data were analyzed by EK, ZT, MT, IHO, OY and FS. UK prepared the manuscript. UK and KS supervised overall project. All authors read and approved the final version of manuscript.
